# Draft Genome Sequence of Heavy Metal-Resistant Bacillus cereus NWUAB01

**DOI:** 10.1128/MRA.01706-18

**Published:** 2019-02-14

**Authors:** Olubukola Oluranti Babalola, Bukola Rhoda Aremu, Ayansina Segun Ayangbenro

**Affiliations:** a Food Security and Safety Niche, Faculty of Natural and Agricultural Sciences, North-West University, Mmabatho, South Africa; University of Southern California

## Abstract

Bacillus cereus NWUAB01 was isolated from a gold-mining site in Vryburg, South Africa, for its multiple heavy metal resistance properties. Here, we report the draft genome sequence of B. cereus NWUAB01 obtained with Illumina sequencing.

## ANNOUNCEMENT

Bacteria from polluted environments have evolved resistance and adaptive genes required for their survival in hostile environments. The search for novel molecular mechanisms from polluted environments provides strong informational resources that lead to new technologies (1). As a result, the draft genome of Bacillus cereus NWUAB01 isolated from soil of a gold-mining site in Vryburg, South Africa (26.1681354 S, 25.246582 E), is reported here.

Bacillus cereus is a spore-forming Gram-positive bacterium that is ubiquitous in nature and always detected in hostile environments (2). The spore-forming ability helps it to survive in different habitats (3). B. cereus NWUAB01 is a metal-resistant spore-forming isolate. Microbial degradation is a major route of recovery of pollutants from polluted environments by transformation and detoxification mechanisms (4). B. cereus NWUAB01 was identified from 98 bacterial isolates from soil samples obtained from a gold-mining site. The collection was screened for heavy metal (Cd, Cr, and Pb) resistance and secondary metabolite production. Soil samples were serially diluted and plated out on Luria-Bertani (LB) agar that had been supplemented with 50 mg/liter of each metal solution and a mixture of all the metals. The metal solutions (CdSO_4_, K_2_CrO_4_, and Pb(NO_3_)_2_) were filter sterilized through 0.22-µm membrane filter (Millipore Corporation, Bedford, MA). The plates were incubated at 30°C for 48 h (5). A pure culture of strain NWUAB01 was screened for its ability to grow on different metal concentrations (100 to 1,000 mg/liter) on LB agar plates.

The genomic DNA of strain NWUAB01 was extracted from a pure culture grown on LB agar with the soil microbe DNA Mini Prep DNA extraction kit (Zymo Research, CA) following the kit’s protocol. The DNA quality and quantity were determined using a NanoDrop Lite spectrophotometer (Thermo Fischer Scientific, CA). The DNA was sent to Inqaba Biotec, a commercial next-generation sequencing (NGS) service provider in South Africa for 16S rRNA and whole-genome sequencing. The draft genome of B. cereus NWUAB01 was obtained with whole-genome shotgun sequencing using an Illumina paired-end library with an average insert size of ∼300 bp. We used 50 ng of the DNA sample to prepare the library with the Nextera DNA sample preparation kit (Illumina). The sample was fragmented with an ultrasonication approach (Covaris). The resulting DNA fragments were size selected (300 to 800 bp) with AMPure XP beads. The fragments were then end repaired, and Illumina-specific adapter sequences were ligated to each fragment. The sample was indexed, and a second size selection step was performed. The sample was then quantified with a fluorometric method, diluted to a standard concentration (4 nM), and then sequenced on Illumina’s MiSeq platform with a MiSeq v3 (600-cycle) kit as described in the kit protocol.

The raw sequence was processed to obtain high-quality reads with the KBase (6) platform. The read quality was checked with FastQC (v.1.0.4) (7), and the reads were trimmed to remove adapter and low-quality sequences and ambiguous reads using Trimmomatic (v0.36) (8) with default parameters. *De novo* assembly was performed with SPAdes v.3.12.0 (9) with default settings to yield 91 contigs. The draft genome sequence of B. cereus NWUAB01 consists of 5,989,415 bp with a G+C content of 35.01%. The contigs have an *N*_50_ value of 326,240 bp and an *L*_50_ value of 7, and the largest contig has 644,886 bp. The contigs were annotated with the Rapid Annotations using Subsystems Technology (RAST v 2.0) server (10) and the NCBI Prokaryotic Genome Annotation Pipeline (PGAP v4.7) (11), which identified 6,306 coding genes, 115 RNA, 3 rRNA operons, and 280 pseudogenes.

The genome annotation showed genes responsible for metal resistance, such as the chromate transport protein (ChrA), cobalt-zinc-cadmium resistance protein (CzcD), copper resistance protein (CopC and CopD), arsenic efflux pump protein, cadmium resistance transporter, and cadmium efflux system accessory protein. Analysis of the secondary metabolite biosynthesis gene clusters with antiSMASH v3.0 (12) with default parameters revealed that B. cereus NWUAB01 has 44 gene clusters involved in antibiotic and secondary metabolite biosynthesis. These include nonribosomal peptide synthetase (NRPS) gene clusters, lipoproteins, lipopolysaccharides, petrobactin, siderophores, binding proteins, bacillibactin, proteins related to the degradation of toxic compounds, and biofilm secretion genes. No clustered regularly interspaced short palindromic repeat (CRISPR) sequences were identified by CRISPRFinder (13). Further investigation will provide additional information about the mechanism involved in the multimetal resistance role of B. cereus NWUAB01 that will facilitate environmental applications.

### Data availability.

This whole-genome shotgun project is deposited at DDBJ/ENA/GenBank under accession number QNGD00000000, BioProject number PRJNA476495, and BioSample number SAMN09435693. The Sequence Read Archive raw reads are deposited under accession number SRR7647568. The version described in this paper is version QNGD03000000.
